# Transplantation of Microglia in the Area of Spinal Cord Injury in an Acute Period Increases Tissue Sparing, but Not Functional Recovery

**DOI:** 10.3389/fncel.2018.00507

**Published:** 2018-12-21

**Authors:** Elvira R. Akhmetzyanova, Yana O. Mukhamedshina, Margarita N. Zhuravleva, Luisa R. Galieva, Alexander A. Kostennikov, Ekaterina E. Garanina, Albert A. Rizvanov

**Affiliations:** ^1^OpenLab Gene and Cell Technologies, Kazan Federal University, Kazan, Russia; ^2^Department of Histology, Cytology and Embryology, Kazan State Medical University, Kazan, Russia

**Keywords:** microglia, spinal cord injury, *egfp*, *gdnf*, adenoviral vector

## Abstract

Microglial cells are known as important mediators of inflammation and immune response in the central nervous system (CNS). However, a neuroprotective role of these cells in post-traumatic processes should not be overlooked. Microglial cells are the first to respond to CNS injury and are further involved in all critical events of pathogenesis. When activated microglia clear the cellular debris and release anti- and proinflammatory cytokines and chemokines, nitric oxide, neurotrophins, and antioxidants capable of producing both neurotoxic and neuroprotective effects. The aim of this study was to determine to what extent the phagocytic activity of microglia in an acute period of spinal cord injury (SCI) in rats can effect the post-traumatic processes. For this purpose we implanted genetically modified Ad5-EGFP or Ad5-GDNF microglial cells into the area of acute SCI. Our experiments demonstrate that the area of intact tissue was lower in the group transplanted with Ad5-GDNF-transduced microglial cells with reduced phagocytic activity than that in the group of animals transplanted with Ad5-EGFP-transduced microglia cells which did not affect the cell activity. At the same time, there was no significant difference in the functional recovery index between these groups. Thus, the increased number of microglia cells with good phagocytic activity in the area of acute SCI may contribute to the improved nervous tissue integrity without a significant effect on the functional recovery within 30 days after injury.

## Introduction

Spinal cord injury (SCI) is characterized by numerous pathologic reactions which involve every cell type of the central nervous system (CNS). It results in neuron and glial cell death, is followed by nerve fiber degeneration, oxidative stress, and other pathological alterations. The activation of microglial cells which are the first to respond to nerve tissue damage is one of the essential events of post-traumatic reactions (Hansson, 2003; Dibaj et al., 2010; Silver et al., 2015). When activated the microglia clear the cellular debris and release anti- and pro-inflammatory cytokines and chemokines, nitric oxide, neurotrophins, and antioxidants capable of exhibiting both neurotoxic and neuroprotective effects (Kreutzberg, 1996; Lai and Todd, 2008; Shechter and Schwartz, 2013). Lately some approaches to SCI treatment are focusing on modulating microglial reactivity after injury toward a neuroprotective phenotype.

Francos-Quijorna et al. (2016) demonstrated that IL-4 facilitated microglia/macrophages gaining the M2 phenotype, promoting nerve tissue regeneration and functional recovery after SCI. M2a macrophages promoted by IL-4, IL-13, and arginase-1 mainly participate in reducing inflammation, enhancing phagocytosis and differentiation of neural stem cells (NSCs) (Varnum and Ikezu, 2012). The introduction of M2 macrophages into the spinal cord and brain is shown to be effective for the treatment of non-infectious inflammation of the CNS (Weber et al., 2007; Shechter et al., 2013). These studies suggest that modulation of microglial cells toward the neuroprotective phenotype seems to be quite promising in order to stimulate neuroregeneration in SCI.

Our present study was based on the use of GDNF as a potent inhibitor of microglial phagocytic activity to promote the expression of neuroprotective or neurotoxic phenotypes (Rocha et al., 2012; Zhuravleva et al., 2016). The enhancement of the scavenger and phagocytic properties of microglia is supposed to result in early resolution of the initial traumatic events that can improve long-term structural and functional outcome (Redondo-Castro et al., 2013). To test this hypothesis we transplanted genetically modified Ad5-EGFP or Ad5-GDNF microglial cells into the area of acute SCI and followed the animals for 5 weeks, assessing functional improvements and changes in tissue integrity.

## Materials and Methods

### Microglia Isolation and Cultivation

Microglia were isolated from neonatal rat cerebral cortex as described previously (Zhuravleva et al., 2015). Briefly, pups were anesthetized and perfused via the left ventricle with cold DPBS solution supplemented with 5 U/ml sodium heparin. The cortex was isolated, and the pia mater peeled off. Tissue was mechanically homogenized and incubated with an enzyme solution (papain 2 mg/ml, DNAse 50 U/ml, dispase 2.5 U/ml) in DPBS for 30 min at 37°C with shaking at 180 rpm. Cells were separated using a 40 μm cell strainer followed by Optiprep (Sigma) discontinuous density gradient for 40 min at 300 g. Layers were cell suspensions in 19% Optiprep, 9% Optiprep, 7% Optiprep, DPBS. A microglia fraction was obtained from a layer, located between fractions 19 and 9%. The microglia were cultured in DMEM/F12 medium supplemented with granulocyte colony-stimulating factor (G-CSF,5 ng/ml, Sigma, United States), 10% FBS, 2 mM L-Glutamine and Penicillin-Streptomycin (PanEco, Russia) in humidified 5% CO2. Resulting cells were transplanted into the area of SCI after 24 h of culture.

### Genetic Modification and Characterization of Microglia

The microglia were transduced with Ad5-EGFP or Ad5-GDNF immediately after the isolation of cells with MOI 40, which provides an optimum level of expression of the transgene and has no cytotoxic effect. The day after transfection, the cells were collected for transplantation. Genetically modified microglia were also analyzed 7 days after transduction by immunocytochemistry (ICC) and flow cytometry (FC) for the detection of the expression of EGFP and GDNF. For ICC, cells were fixed in 4% buffered formalin, washed with 0.1% Triton X-100 in PBS and stained with anti-GDNF antibodies (ab10835, Abcam) at a working dilution of 1:100 for 1 h at RT, washed and stained with Alexa Fluor 647 conjugated anti-goat secondary antibodies A-21447 (Invitrogen) for 30 min at RT. The nuclei were stained with DAPI (d9564, Sigma) for 5 min. The results were analyzed using an AxioObserver Z1 (Carl Zeiss). Briefly, for FC microglial cultures were trypsinized, prefixed with CytoFix (BD Biosciences) and permobilized using Perm 2 (BD Biosciences), incubated with anti-GDNF antibodies (ab10835, Abcam) at a working dilution of 1 μl per 200,000 cells for 30 min at 4°C, washed using centrifugation, stained with Alexa Fluor 555 conjugated anti-goat secondary antibodies (A-21432, Invitrogen) and analyzed using flow cytometer FACS Aria III (BD Biosciences). Ad5-GFP transduced cells were analyzed using FC immediately after trypsinization.

We also analyzed the phenotype of transduced microglia at Day 1, 7, and 14 cultivations. For FC microglia cultures were trypsinized and incubated with antibodies CD11b (101216, BioLegend), CD16 (ab203883, Abcam), CD 40 (ab13545, Abcam), CD 45 (1611525, Sony), CD86 (305420, BioLegend), CD163 (ab182462, Abcam), CD 200 (ab203887, Abcam), CD206 (2205595, Sony) at a working dilution of 1 μl per 200,000 cells for 30 min at 4°C. For CD16, CD40, CD163, and CD200 staining appropriate Alexa Fluor 555 conjugated anti-rabbit secondary antibodies (A-31572, Invitrogen, United States) were used. Stained cells were fixed with CytoFix and analyzed using flow cytometer FACS Aria III. The *in vitro* data were obtained from five independent experiments.

### Spinal Cord Injury and Cell Transplantation

All animal protocols were approved by the Kazan Federal University Animal Care and Use Committee (Permit Number: 2 dated on May 5, 2015). Adult male and female Wistar rats (weight of 250–300 g each; Pushchino Laboratory, Russia) were group housed in clear plastic cages (12 h:12 h light/dark cycle) with food and water available *ad libitum*.

Rats were deeply anesthetized by intraperitoneal injection of chloral hydrate (80 mg/ml, 0.4 ml per 100 g, Sigma). After skin incision, the Th8 vertebra was removed by laminectomy. The impact rod (diameter 2 mm, 10 g) of an impactor was centered above Th8 and dropped from a height of 25 mm to induce SCI. Immediately after SCI, 5 μl suspensions of MG+Ad5-GDNF (n = 15) or MG+Ad5-EGFP (*n* = 15) (containing 1 × 10^6^ cells) were injected into the area of SCI (epicenter) at a depth of 1 mm by using a 5-μl Hamilton syringe. In the control group (SCI) (*n* = 15), 5 μl 0.9% NaCl were injected into the aforementioned point. After SCI the dorsal back musculature and the skin were sutured. Following surgery the rats received doses of gentamicin (25 mg/kg, Omela, Russian Federation) intramuscularly for seven consecutive days. Bladders of injured rats were manually emptied twice a day until spontaneous voiding occurred. There was a control group of 10 intact animals.

### Behavioral Test

Locomotor recovery was assessed in an open field by using the BBB rating scale. The baseline was obtained three days before SCI. To evaluate differences in functional recovery, a behavioral assessment in all groups was performed before SCI, on day 7, and then every second day. Locomotion was scored simultaneously by two observers who were blinded to the treatment groups. Final scores were obtained by averaging the two scores awarded by the examiners.

### Histological Assessment

For histology and immunohistochemistry, 30 days after cell transplantation the rats were anesthetized with chloral hydrate, prior to intracardiac perfusion with 4% paraformaldehyde (PFA, Sigma). After incubation in 30% sucrose, samples were embedded in a tissue freezing medium. Non-fixed tissue was used for RT-PCR. Twenty micrometer transverse tissue sections, obtained with a Microm HM 560 Cryostat, were stained with Azur-eosin for visualizing tissues. Images were captured using a × 20 objective lens and a microscope (APERIOCS2, Leica). The cross-sectional areas of the spared tissue and abnormal cavities were measured on transverse sections of the spinal cord within the midpoint of the lesion center (the epicenter) and in spinal segments 1–5 mm rostral and caudal to the site of injury. The total area of abnormal cavities in the spinal cord cross-section was calculated by adding cavities with an area of not less than 1.500 μm^2^. An Aperio imagescope was used to measure the tissue area.

### Immunofluorescence Analysis

For immunofluorescence labeling, the sections were blocked with 5% normal goat serum for 1 h at room temperature (RT) and then incubated separately overnight at 4°C with a primary antibody against ionized calcium binding adaptor molecule 1 (Iba1) (Abcam, 1:300) and glial fibrillary acidic protein (GFAP) (Millipore, 1:200). Prior to visualization, the sections were incubated with a fluorophore-conjugated secondary antibody (anti- mouse IgG conjugated with Alexa 546, Invitrogen, 1:200) for 2 h at RT. 4′,6-Diamidino-2-phenylindole (DAPI) (10 μg/mL in PBS, Sigma) was used to visualize nuclei. Coverslips were mounted on slides using a mounting medium (ImmunoHistoMount, Santa Cruz). The sections were examined under a LSM 780 Confocal Microscope (Carl Zeiss, Germany). The total intensity of labeling (semi-quantitative analysis of Iba1 and GFAP) was analyzed using Zen 2012 Software (Carl Zeiss). All sections were imaged in the z-plane using identical confocal settings (laser intensity, gain, and offset). Measurements were obtained from transverse histological sections collected at 5-mm increments extending from the contusion center (observed area, 2 mm^2^) of the SCI. The following areas were selected for semiquantitative immunohistochemical evaluation of glial cells: the main corticospinal tract (CST), ventral funiculi (VF), and the ventral horn (VH).

### RNA Isolation and Real-Time PCR Analysis

Total RNA from fresh spinal cords (5 mm long segment encompassing the injury site) was isolated using a Yellow Solve Kit (Silex, Russia) according to the manufacturer’s recommendations. First strand cDNA synthesis was held using 100 U of RevertAid reverse transcriptase (Thermo Fisher Scientific), 100 pmol of random hexamer primers and 5 U of RNAse inhibitor according to the standard protocol. Quantitative analysis of mRNA of *egfp*, *gdnf*, *irf5*, *iba1* genes was applied using a CFX 96 Real-Time PCR System (Bio-Rad, Hercules, CA, United States). Each PCR reaction contained 100 ng cDNA, 2.5× Reaction mixture B (Syntol, Russia), 200 nM of each primer, and the probe (100 nM) (Table 1). The mRNA expression was normalized according to the 18S RNA transcription. To create standard curves plasmid DNA with corresponding inserts was used. The level of mRNA in non-treated spinal cord after injury was considered as 100%.

**Table 1:** Primers and probes for RT-PCR.

Primer	Nucleotide sequence
18S-TM-Forward	gCCgCTAgAggTgAAATTCTTg
18S-TM-Reverse	CATTCTTggCAAATgCTTTCg
18S-TM-Probe	[HEX]ACCgCgCAAgACggACCAg[BH2]
EGFP-TM-Forward	AgCAAAgACCCCAACgAgAA
EGFP-TM-Reverse	ggCggCggTCACgAA
EGFP-TM-Probe	[FAM]CgCgATCACATggTCCTgCTgg[BH1]
GDNF-TM-Forward	CgCTgAgCAgTgACTCAAAT
GDNF-TM-Reverse	CgATTCCgCTCTCTTCTAgg
GDNF-TM-Probe	[FAM]TCCATgACATCATCgAACTgATCAgg[BH1]
Irf5-TM-Forward	AgggCTTCAATgggTCAAC
Irf5-TM-Reverse	gTgTATTTCCCTgTCTCCTTgg
Irf5-TM-Probe	[HEX]ATggTgTTATCTCCgTCCTggCTg[BH2]
Iba1-TM-Forward	ACCAgCgTCTgAggAgCTAT
Iba1-TM-Reverse	AggAAgTgCTTgTTgATCCC
Iba1-TM-Probe	[HEX]CCCTgCAAATCCTTgCTCTggC[BH2]

### Statistical Analysis

All statistical analyses were carried out by two independent biostatisticians blinded to the groups. Data are presented as means ± standard deviation. The one-way analysis of variance (ANOVA) with the Tukey’s test or two-way analysis of variance (ANOVA) were used for multiple groups. Values of *P* < 0.05 and *P* < 0.01 were considered statistically significant. Data were analyzed using the Origin 7.0 SR0 Software (OriginLab, Northampton, MA, United States).

the expression of EGFP and GDNF *in vitro* and *in vivo* in the area of SCI. Immunocytochemistry results demonstrate expression GDNF **(A,C)** and EGFP **(B,D)** in transduced by GDNF **(C)** or EGFP **(B)** and non-transduced/native **(A,D)** microglia. Results of flow cytometry are shown **(E,F)**. Based on the flow cytometry data, 51% of the transduced by Ad5-EGFP microglia were EGFP-positive (red color in dot plot, yellow color is for non-transduced cells) **(E)** and 50% of the transduced by Ad5-GDNF microglia demonstrated GDNF overexpression (red color on dot plot, yellow color in dot plot for native cells, gray color is for unstained cells) **(F)**. EGFP and GDNF mRNA expression *in vitro* on cultivation Days 7 **(G)** and *in vivo* at Day 30 after SCI in experimental groups **(H,I)**. The EGFP and GDNF mRNA expression levels in the SCI and Intact controls groups, respectively, were considered 100% **(H,I)**. ^∗^*P* < 0.05, ^∗∗^*P* < 0.01, one-way ANOVA followed by a Tukey’s *post hoc* test. Ad5-EGFP microglia around the small cavities (asterisks) **(J,K)** and in ventral horns **(L)** at the distance of 3 mm in rostral and 3–4 mm in caudal directions from the injury epicenter, respectively, at Day 30 after SCI and transplantation. Nuclei are stained with DAPI (blue). Scale bar: 20 **(J,K)** and 10 **(L)** μm.

**Table 2:** Results of flow cytometry in microglia culture.

Markers	1 day MG+Ad5-EGFP	1 day MG+Ad5-GDNF	7 day MG+Ad5-EGFP	7 day MG+Ad5-GDNF	14 day MG+Ad5-EGFP	14 day MG+Ad5-GDNF
CD11b	30 ± 5%	25 ± 3%	30 ± 5,5%	23 ± 1,5%	18,5 ± 1,5%	26 ± 3,5%
CD16	98 ± 0,5%	94 ± 3,5%	45 ± 3%	60 ± 5,5%	11 ± 0,5%	11 ± 1,5%
CD40	99 ± 0,5%	98 ± 0,5%	36 ± 4,5%	22 ± 2,5%	11 ± 1,5%	8 ± 0,5%
CD45	86 ± 5%	83 ± 3%	45 ± 4,5%	40 ± 2,5%	15,5 ± 1,5%	1 ± 0,5%^*^
CD86	99 ± 0,5%	99 ± 0,5%	96 ± 0,5%	94 ± 1,5%	99 ± 0,5%	92 ± 0,5%
CD163	84 ± 5%	76 ± 3,5%	30 ± 2,5%	30 ± 2%	11,5 ± 0,5%	8 ± 0,5%
CD200	98 ± 1%	93 ± 2,5%	40 ± 2,5%	30 ± 3,5%	10 ± 0,5%	10 ± 1%
CD206	35 ± 1,5%	35 ± 1%	42 ± 2,5%	35 ± 5,5%	14 ± 1,5%	13 ± 1%

*^∗^P < 0.05, compared to MG+Ad5-EGFP in the relevant period.*

## Results

### Analysis of the Expression of EGFP and GDNF *in vitro* and *in vivo* in the Area of SCI

**FIGURE 1:**
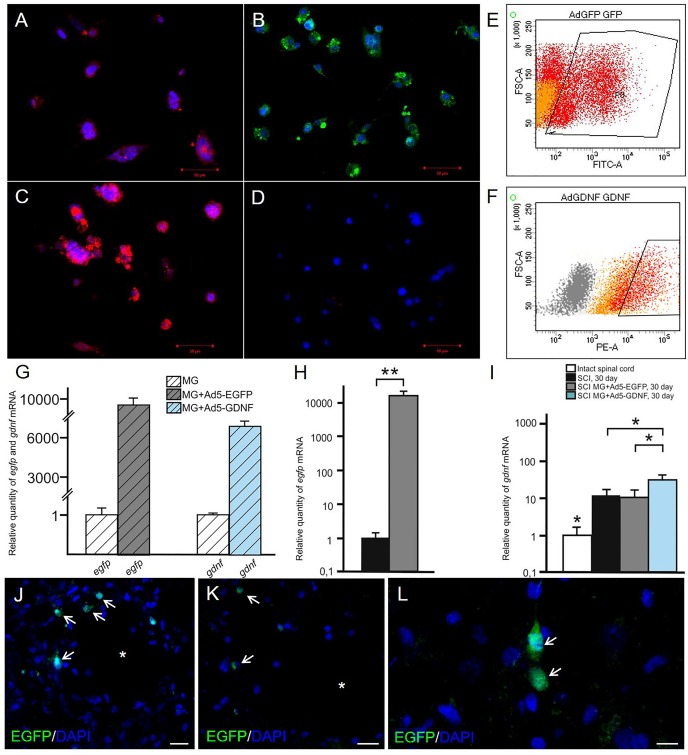
Analysis of

Seven days after microglia transduction with Ad5-EGFP or Ad5-GDNF, EGFP, and GDNF expression was studied *in vitro* (Figure 1). ICC and FC demonstrated that native microglia expressed GDNF. On day 7 after Ad5-GDNF transduction 50% of microglia demonstrated GDNF overexpression (Figures 1A,C,F). Visualized cells by fluorescent microscopy had green fluorescence (MG+Ad5-EGFP) compared to non-transduced/native microglia (Figures 1B,D). On day 7 after transduction, 51% of the microglial cells expressed EGFP (Figure 1E). mRNA expression in microglia immediately treated with Ad5-EGFP or Ad5-GDNF was 9689 and 6733 times higher relative to non-transduced cells, respectively, at Day 7 after transduction (Figure 1G).

We also studied to what extent the transplantation of genetically modified microglia increased the expression of EGFP and GDNF mRNAs in the area of SCI. Thus on day 30 after SCI EGFP mRNA was more than 10000 times higher in the spinal cord of SCI MG+Ad5-EGFP rats than in untreated rats subjected to SCI (Figure 1H). The results also demonstrated that the GDNF mRNA expression level was significantly higher in the group with MG-GDNF transplantation as compared to the other experimental groups (*P* < 0.01) (Figure 1I). The lowest expression level was observed in the intact spinal cord, with no reliable difference detected between SCI groups without cell transplantation and those transplanted with MG+Ad5-EGFP. Thus, the data obtained for EGFP and GDNF mRNAs levels in the area of SCI confirmed the efficacy of target gene delivery and possible long-term expression of recombinant gene products.

### Distribution and Survival of Transplanted Microglia

Previously in similar experimental conditions, we showed, that MG+Ad5-EGFP survived in the early period of SCI (not less than 14 days), actively expressed recombinant gene *egfp* and migrated up to 5 and 3 mm in the rostral and caudal directions, respectively, from the injection site (Zhuravleva et al., 2015). At Day 30 we observed EGFP^+^-cells arranged predominantly around the small cavities at the distance of 3–4 mm in both directions from the injury epicenter (Figures 1J,K) and up to 1–2 EGFP^+^-cells in ventral horns in similar directions (Figure 1L).

### Analysis of Changes in Phenotype and Gene Expression of Microglia *in vitro*

Previously, we showed that 2 week cultured microglia (non-transduced cells) express Iba1, CD68, CD11b/c, and CD45 (Zhuravleva et al., 2015). Microglia had an amoeboid shape initially, but after 2 weeks branched forms of microglia were seen as well. In this study, we estimated the expression level of the following markers (CD11, CD16, CD40, CD45, CD86, CD163, CD200, CD206) in the obtained cultures of transduced microglia at Days 1, 7, and 14 (Table 2). At Day 1 we observed maximal levels expression of CD16, CD40, CD86, and CD200 in both culture of transduced microglia without any significant difference in all 8 markers. At Day 7 we observed a significant decline of the expression of CD16, CD40, CD45, CD163, and CD200 without significant differences between microglia transduced by Ad5-EGFP or Ad5-GDNF. Expression of CD11 and CD206 was similar at Day 1 and 7. CD86 only showed consistently high expression levels (more 90%) after 14 days compared to other markers which declined significantly during that time. We observed the only significant difference between two types of transduced microglia for CD45, which had a more than 15-fold increase in microglia transduced by Ad5-EGFP.

**FIGURE 2:**
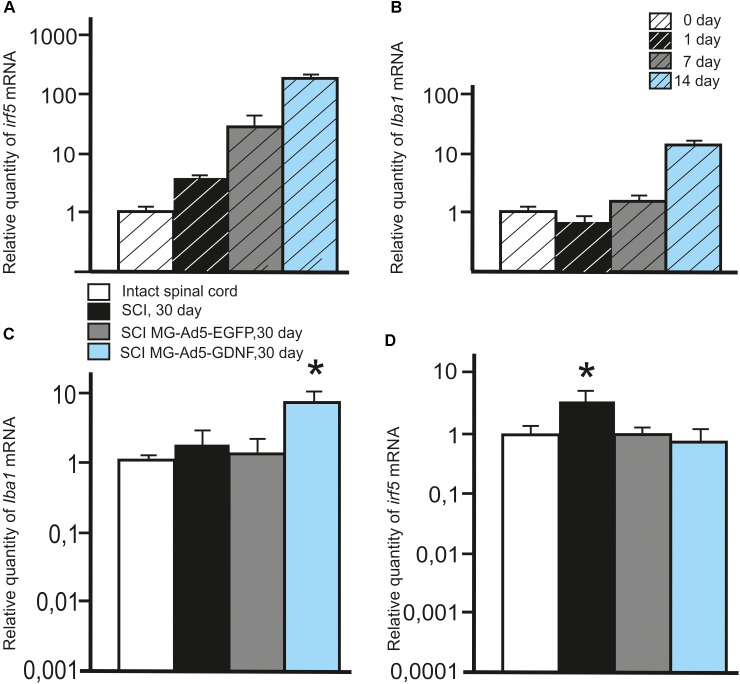
Analysis of the expression of Iba1 and Irf5 mRNA *in vitro* and *in vivo* in the area of SCI. Irf5 **(A)** and Iba1 **(B)** mRNA expression *in vitro* on cultivation Days 0, 1, 7, 14. Irf5 and Iba1 mRNA expression of 0 day group was considered 100%. Differences were statistically significant between all time intervals (*P* < 0.05). Iba1 **(C)** and irf5 **(D)** mRNA expression *in vivo* on day 30 after SCI and cell transplantation. The Iba1 and irf5 mRNA expression levels in Intact controls were considered 100% **(C,D)**. ^∗^*P* < 0.05, one-way ANOVA followed by a Tukey’s *post hoc* test.

We also investigated the expression of pan marker Iba1 and M1 phenotype marker irf5 mRNAs in the microglia at the same time points. The analysis showed that the maximum expression of both Iba1 and irf5 mRNAs was observed on day 14. The irf5 expression gradually increased from 0 to 14 days (*P* < 0.05) (Figure 2A). By culture day 1 the Iba1 expression was below that at the time of isolation (*P* < 0.05); however, this parameter consistently increased by culture day 14 (*P* < 0.05) (Figure 2B).

### Assessment of Microglia in the Area of SCI

To investigate the expression of Iba1 and irf5 mRNAs in injured spinal cord, qRT-PCR was performed on sections obtained from the injury site 30 days post transplantation. When evaluating the Iba1 and irf5 mRNAs levels specific for microglial cells/macrophages, we detected the following pattern in the area of SCI. The Iba1 mRNA level was significantly higher in the group with MG-GDNF transplantation as compared to the other experimental groups. At the same time there was no significant difference in the Iba1 mRNA expression between intact spinal cord group with SCI and transplantation of MG-EGFP (*P* < 0.01) (Figure 2C). When evaluating the expression of irf5 mRNA, this value was significantly higher in the SCI group without cell transplantation (*P* < 0.01), than in the other experimental groups, with no reliable differences between them (Figure 2D).

**FIGURE 3:**
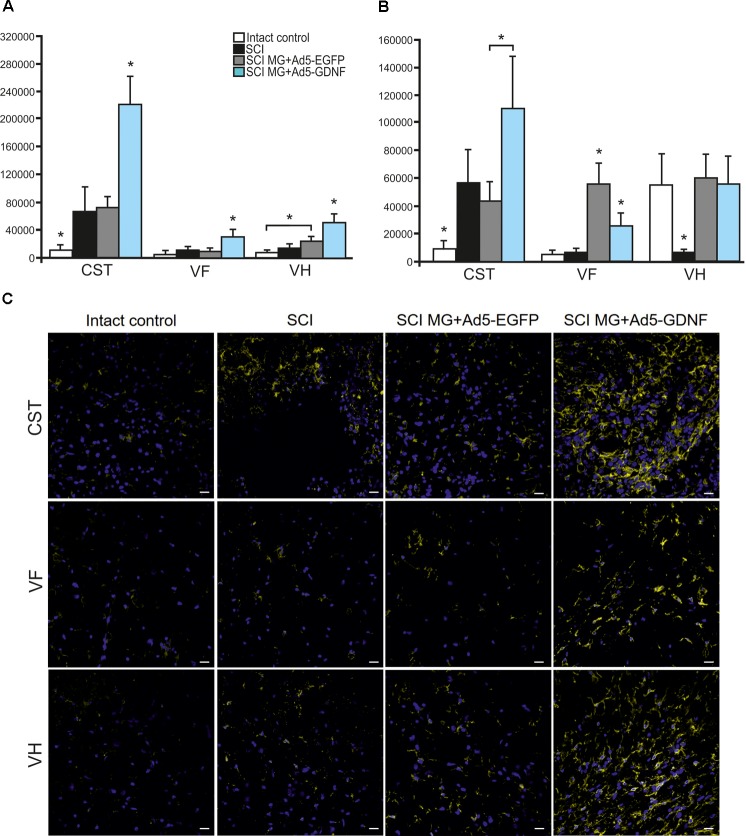
Assessment of microglial cells in the area of SCI. The total intensity of Iba1 labeling 5 mm rostrally **(A)** and caudally **(B)** from the injury epicenter. ^∗^*P* < 0.05, one-way ANOVA followed by a Tukey’s *post hoc* test. Assessment of microglial cells with Iba1 5 mm rostrally from the injury epicenter within the CST, VF and VH in the experimental groups **(C)**.

Using immunofluorescence we studied the total intensity of Iba1 labeling 5 mm rostrally and caudally from the injury epicenter in selected zones of the white and gray matter. It was significantly higher rostrally from the injury epicenter in all study zones in the group with MG+Ad5-GDNF transplantation (*P* < 0.05) (Figure 3A). At the same time, there was no significant difference in the total intensity of Iba1 labeling in the SCI and SCI MG+ Ad5-EGFP groups. A maximum Iba1^+^-cell concentration was detected in the CST zone of the SCI MG+Ad5-GDNF group in both directions from the site of injury (Figures 3A–C). A minimal Iba1^+^microglial cell concentration was identified in the VF zone (in both directions from the site of injury) in the intact group (Figure 3C).

Caudally from the injury epicenter the total intensity of Iba1 labeling in VF and VH zones was higher (*P* < 0.05) in the groups with cell transplantation as compared to the SCI group without cell transplantation. Moreover, this parameter in VF zone was significantly higher (twofold) in the group with MG+Ad5-EGFP transplantation as compared to that transplanted with MG+Ad5-GDNF (*P* < 0.05).

### Tissue Sparing

**FIGURE 4:**
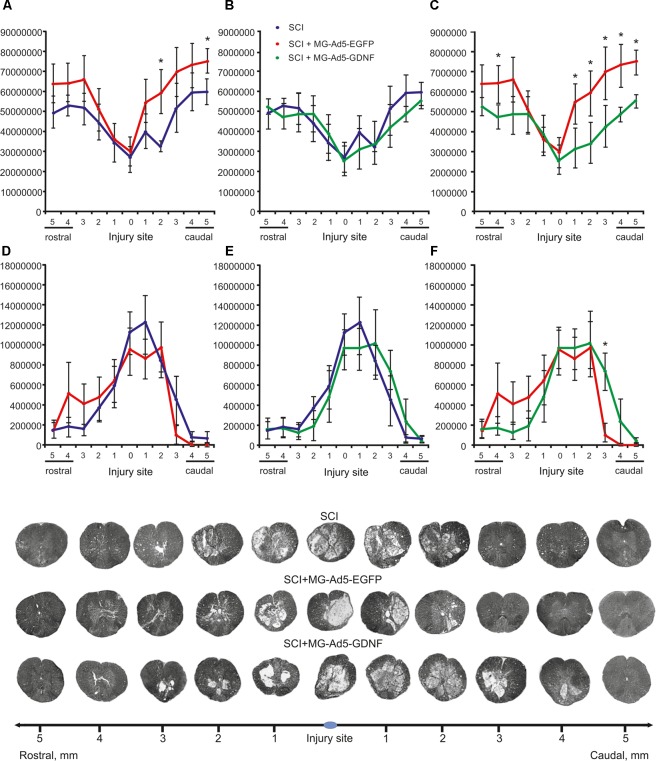
Tissue analysis in experimental groups. An area of the intact tissue **(A–C)** and a total area of abnormal cavities **(D–F)** 5 mm rostrally and caudally from the injury epicenter on Day 30 after SCI and microglia transplantation. ^∗^*P* < 0.05, one-way ANOVA followed by a Tukey’s *post hoc* test. Cross sections of the injured spinal cord on Day 30 after SCI in experimental groups (bottom panel). Azur-eosin staining.

Morphometry allowed us to identify differences in the intact tissue area and a total area of abnormal cavities up to 5 mm rostrally and caudally from the injury epicenter in all study groups (Figure 4). This analysis demonstrated that within the study range the area of intact tissue was greatest in the SCI MG+Ad5-EGFP group. There was a significant difference at a distance of 2 (>1.85-fold) and 5 mm (>1.26-fold) caudally as compared to the SCI group without cell transplantation (Figure 4A) and at a distance of 4 mm (>1.35-fold) rostrally and 1–5 mm (>1.34 to 1.73-folds) caudally as compared to the SCI MG+Ad5-GDNF group (Figure 4C). At the same time no significant differences were observed between SCI and SCI MG+Ad5-GDNF groups (Figure 4B).

The total area analysis of abnormal cavities revealed no reliable differences between experimental groups (Figures 4D–F). There was an only exception at a distance of 3 mm caudally where this value was sevenfold lower in the SCI MG+Ad5-EGFP group than that in the SCI MG+Ad5-GDNF one (Figure 4F).

Thus, the morphometric analysis demonstrated that at every distance studied the intact tissue area was greater in the SCI MG+Ad5-EGFP group than in the SCI group without cell transplantation and the SCI MG+Ad5-GDNF group. However, it should be noted that this significant difference was observed in the caudal direction. There was a significant difference in the area of abnormal cavities between SCI MG+Ad5-EGFP and SCI MG+Ad5-GDNF groups in the caudal direction as well.

### Assessment of Astrocytes in the Area of SCI

**FIGURE 5:**
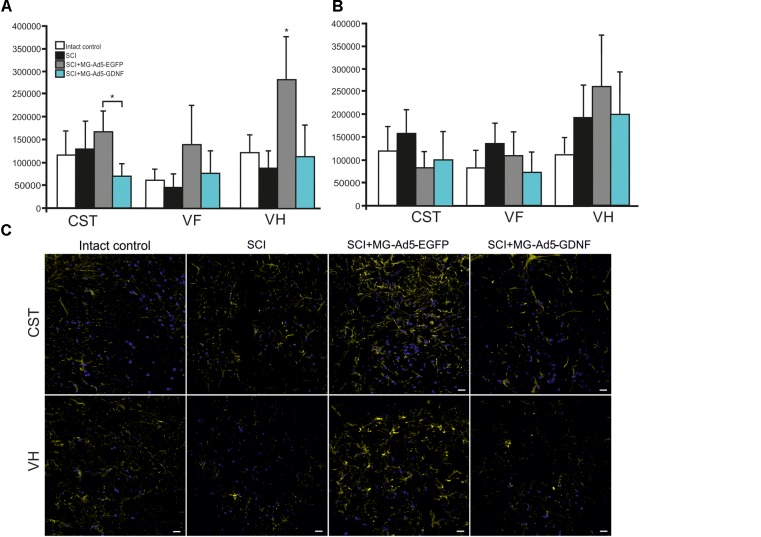
Assessment of astrocytes in the area of SCI. The total intensity of GFAP labeling 5 mm rostrally **(A)** and caudally **(B)** from the injury epicenter. ^∗^*P* < 0.05, one-way ANOVA followed by a Tukey’s *post hoc* test. Assessment of astrocytes with GFAP 5 mm rostrally from the injury epicenter within the CST and VH in the experimental groups **(C)**.

Using the method of immunohistochemistry we studied the total intensity of GFAP labeling 5 mm rostrally and caudally from the injury epicenter in selected zones of the white and gray matter (Figures 5A,B). 5 mm rostral to the injury epicenter the GFAP fluorescence intensity was highest in all studied zones in the group with MG+Ad5-EGFP transplantation (Figure 5C). This value was 2-fold higher in CST and VH zone (*P* < 0.05) in the SCI MG+Ad5-EGFP group as compared to the SCI MG+Ad5-GDNF one. No significant differences between SCI and SCI MG+Ad5-GDNF groups were observed both rostrally and caudally from the injury epicenter. At the same time, there was a significant difference in the VH zone at a distance of 5 mm rostrally where the total intensity of GFAP labeling was ∼twofold (*P* < 0.05) higher in the SCI MG+Ad5-EGFP group than that in other experimental groups.

### Assessment of Locomotor Activity

**FIGURE 6:**
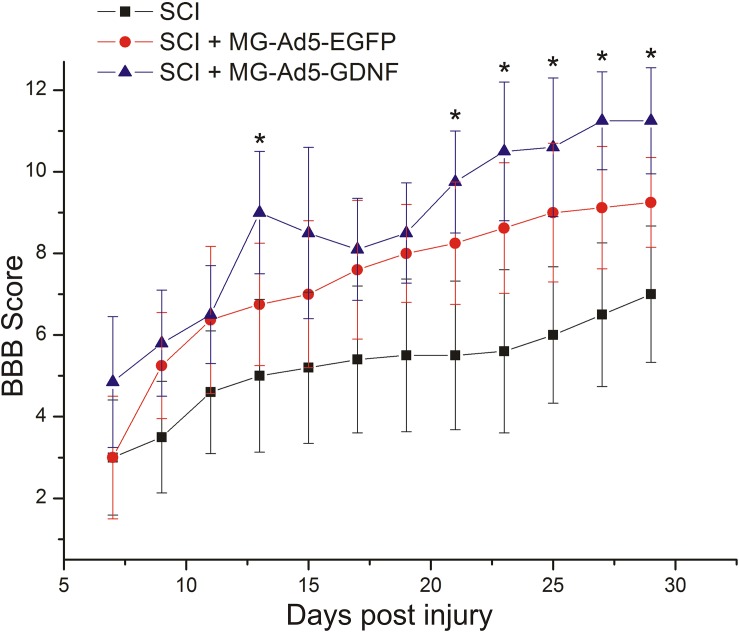
Post-SCI behavioral studies in experimental groups. Assessment of locomotor activity using the BBB rating scale from 7 to 30 days after injury. ^∗^*P* < 0.05, one-way ANOVA followed by a Tukey’s *post hoc* test.

We assessed locomotor recovery using the BBB rating scale from 7 to 30 days post injury. The motor function scores in the SCI+MG-Ad5-GDNF group were higher than those in the SCI and SCI+MG-Ad5-EGFP groups within 30 days after injury (Figure 6). Additionally, there were significant differences (*P* < 0.05) only between the SCI+MG-Ad5-GDNF and SCI groups on Days 14 and 21–30 after injury. We observed no significant differences between SCI and SCI+MG-Ad5-EGFP groups as well as between SCI+MG-Ad5-EGFP and SCI+MG-Ad5-GDNF ones.

## Discussion

In our previous study, we showed that glial cell line-derived neurotrophic factor (GDNF), a well-known neuroprotective molecule, decreases phagocytic activity of microglia in an *in vitro* model of SCI (Zhuravleva et al., 2016). Ad5-GDNF transducted microglia have shown a similar effect. Therefore, we decided to identify to what extent the microglia phagocytic activity can affect the outcome of post-traumatic processes in the acute period of SCI in rats. Our experiments demonstrated that the intact tissue area was lower in the group with Ad5-GDNF-transduced microglial cell transplantation and had reduced phagocytic activity than that in the group of animals transplanted with microglial cells carrying the gene *egfp*. Interestingly, there were no significant differences in restoring the motor function.

We have previously characterized the microglial transplanted cells (Zhuravleva et al., 2015). Immunocytochemistry demonstrated that immediately after isolation and within 2 weeks of culture the microglial cells expressed Iba1, CD68, CD11b/c, and CD45. In the same time, our results demonstrate that most of the transduced Ad5-EGFP well as Ad5-GDNF microglia predominantly express pan markers Iba1/CD68 and CD86 at all studied culture periods. In their morphologic properties Ad5-EGFP transducted microglia were amoeboid active phagocytic microglia. Amoeboid morphology was shown to reflect a highly active state associated with phagocytosis and a proinflammatory function (Tam and Ma, 2014).

At the same time transduction of microglia with Ad5-GDNF promoted the formation of round, resting non-phagocytic microglia. These findings are in line with the conclusions that GDNF can suppress activation of the microglia in neuroinflammatory processes (Xing et al., 2010; Rickert et al., 2014). Our findings for transplantation effects of active phagocytic and non-phagocytic microglia into the area of SCI are consistent with the assumption made by Redondo-Castro et al. (2013) that the enhancement of the scavenger and phagocytic properties of microglia resulted in was reduction of initial trauma which can improve long-term structural and functional outcome. This positive effect of active phagocytic microglia is due to the removal of dead cells and debris (Boekhoff et al., 2011). Yaguchi et al. (2008) demonstrated that activation of endogenous microglia after SCI improved the integrity of nervous tissue. Moreover, these results are relevant not only to an acute period of injury but also to a chronic one. There is a significant reduction of scar tissue (Hamanoue et al., 2015) with activation of endogenous microglia in the chronic post-traumatic period.

We have previously shown that the microglia transplanted into the area of SCI in an acute period actively migrated rostrally and caudally from the site of injection and survived for at least 14 days with the expression of reporter genes (Zhuravleva et al., 2016). Throughout the whole period of microglia cultivation, the level of Iba1 and irf5 mRNA expression was increased with the maximum value observed on 14 day. This result corresponds to the data that G-CSF (which was added to the culture of microglia) promotes the division of microglia and the maintenance of its functional state (Giulian and Ingeman, 1988). The results obtained in this study provide evidence to the good survival of the microglia implanted into the site of injury by the expression of Iba1 and irf5 mRNA in the area of SCI at 30 dpi. We demonstrated that the level of irf5 mRNA expression was significantly higher in the SCI group. This result is consistent with the data that after SCI the pool of M1 microglia is increasing (Kigerl et al., 2009) along with irf5 expression (Lawrence and Natoli, 2011). At the same time the level of mRNA Iba1 was significantly higher in groups with microglia transplantation when compared to the other groups. These results seem to be associated with the large number of microglia within the site of injury due to insertion of a donor cell pool in transplantation. It should be noted that the level of mRNA Iba1 (as a pan marker of microglia) was significantly higher in the group with MG-Ad5-GDNF transplantation. The immunohistochemical findings confirmed the results obtained in a real-time PCR analysis and demonstrated a significantly increased Iba1^+^-cell count predominantly in the rostral direction in the group with MG-Ad5-GDNF transplantation as compared to the other experimental groups. This may be attributed to a supportive GDNF effect on microglial carrier-cells. This is consistent with the previous findings by Salimi et al. (2003) that GDNF promotes cell survival in serum-free cultures of primary rat microglia.

At the same time, immunohistochemical staining for GFAP demonstrated that in most cases an increased intensity of the Iba1 fluorescence was accompanied with decreased intensity of the GFAP fluorescence and vice versa, in the groups with microglia transplantation. This is especially obvious at a distance of 5 mm rostrally where the total intensity of GFAP labeling in all studied zones was higher in the group with MG-Ad5-EGFP transplantation as compared to the SCI+MG-Ad5-GDNF group. Based on these findings it can be concluded that the more microglia in the site of injury, the fewer the number of reactive GFAP^+^-astrocytes is. The data obtained are consistent with the results of Hamanoue et al. (2015), who demonstrated that activated endogenous microglia within a chronic period alter SCI could lead to significant reduction of scar tissue. Nevertheless, the glial barrier and reactive astrocytes have a positive role in maintaining the structural integrity of spinal cord tissue (Adams and Gallo, 2018). In this regard, an increased astroglial activation can be involved in tissue sparing in the group with MG-Ad5-EGFP transplantation.

We found out that transplantation of MG-Ad5-GDNF into the area of SCI improved recovery of the motor function compared to the SCI group. Nevertheless, there were no significant differences between the groups with microglial transplantation. Higher BBB test scores in the group with MG-Ad5-GDNF transplantation might be due to a supportive effect of GDNF on intact axons and their myelinati; the neuroprotective action maintaining the expression of neurofilament proteins, calcitonin gene-related peptide (CGRP) and axon growth associated protein 43 (GAP-43) (Cheng et al., 2002; Zhang et al., 2009).

Thus, the increased number of active phagocytic microglia in the area of acute SCI might promote improved nerve tissue integrity without a significant effect on functional recovery within 30 days after injury.

## Author Contributions

YM: conceived and designed the experiments, behavioral study, statistical analysis, and writing the article. EA and MZ: isolation and preparation of rat microglia, histological assessment and immunohistochemical studies. LG: SCI and cell injection. EG: RNA isolation, cDNA synthesis, and real-time PCR. AK: SCI and post-surgical care, behavioral study. AR: development of a research plan and participation in the writing of the article.

## Conflict of Interest Statement

The authors declare that the research was conducted in the absence of any commercial or financial relationships that could be construed as a potential conflict of interest.

## References

[B1] Adams K. L. Gallo V. (2018). The diversity and disparity of the glial scar. *Nat. Neurosci.* 21 9–15. 10.1038/s41593-017-0033-9 29269757 PMC5937232

[B2] Boekhoff T. M. Ensinger E. M. Carlson R. Bock P. Baumgartner W. Rohn K. (2011). Microglial contribution to secondary injury evaluated in a large animal model of human spinal cord trauma. *J. Neurotrauma* 29 1000–1011. 10.1089/neu.2011.1821 21599492

[B3] Cheng H. Wu J. P. Tzeng S. F. (2002). Neuroprotection of glial cell line-derived neurotrophic factor in damaged spinal cords following contusive injury. *J. Neurosci. Res.* 69 397–405. 10.1002/jnr.10303 12125080

[B4] Dibaj P. Nadrigny F. Steffens H. Scheller A. Hirrlinger J. Schomburg E. D. (2010). NO mediates microglial response to acute spinal cord injury under ATP control in vivo. *Glia* 58 1133–1144. 10.1002/glia.20993 20468054

[B5] Francos-Quijorna I. Amo-Aparicio J. Martinez-Muriana A. López-Vales R. (2016). IL-4 drives microglia and macrophages toward a phenotype conducive for tissue repair and functional recovery after spinal cord injury. *Glia* 64 2079–2092. 10.1002/glia.23041 27470986

[B6] Giulian D. Ingeman J. E. (1988). Colony-stimulating factors as promoters of ameboid microglia. *J. Neurosci.* 8 4707–4717. 3058881 10.1523/JNEUROSCI.08-12-04707.1988 PMC6569554

[B7] Hamanoue M. Morioka K. Hayakawa K. Nakajima K. Ogata T. Takamatsu K. (2015). “Functional recovery from chronic spinal cord injury by the reactivation of endogenous microglia,” *Proceedings of the Neuroscience 2015 Chicago Nanosymposium, Spinal Cord, Therapeutic Strategies*, Chicago, IL.

[B8] Hansson E. (2003). Glial neuronal signaling in the central nervous system. *FASEB J.* 17 341–348. 10.1096/fj.02-0429rev 12631574

[B9] Kigerl K. A. Gensel J. C. Ankeny D. P. Alexander J. K. Donnelly D. J. Popovich P. G. (2009). Identification of two distinct macrophage subsets with divergent effects causing either neurotoxicity or regeneration in the injured mouse spinal cord. *J. Neurosci.* 29 13435–13444. 19864556 10.1523/JNEUROSCI.3257-09.2009 PMC2788152

[B10] Kreutzberg G. W. (1996). Microglia: a sensor for pathological events in the CNS. *Trends Neurosci.* 19 312–318. 10.1016/0166-2236(96)10049-7 8843599

[B11] Lai A. Y. Todd K. G. (2008). Differential regulation of trophic and proinflammatory microglial effectors is dependent on severity of neuronal injury. *Glia* 56 259–270. 10.1002/glia.20610 18069670

[B12] Lawrence T. Natoli G. (2011). Transcriptional regulation of macrophage polarization: enabling diversity with identity. *Nat. Rev. Immunol.* 11:750. 10.1038/nri3088 22025054

[B13] Redondo-Castro E. Hernández J. Mahy N. Navarro X. (2013). Phagocytic microglial phenotype induced by glibenclamide improves functional recovery but worsens hyperalgesia after spinal cord injury in adult rats. *Eur. J. Neurosci.* 38 3786–3798. 10.1111/ejn.12382 24112298

[B14] Rickert U. Grampp S. Wilms H. Spreu J. Knerlich-Lukoschus F. Held-Feindt J. (2014). Glial cell line-derived neurotrophic factor family members reduce microglial activation via inhibiting p38MAPKs-Mediated inflammatory responses. *J. Neurodegener. Dis.* 2014 1–10. 10.1155/2014/369468 26317008 PMC4437344

[B15] Rocha S. M. Cristovão A. C. Campos F. L. Fonseca C. P. Baltazar G. (2012). Astrocyte-derived GDNF is a potent inhibitor of microglial activation. *Neurobiol. Dis.* 47 407–415. 10.1016/j.nbd.2012.04.014 22579772

[B16] Salimi K. Moser K. V. Marksteiner J. Reindl M. Humpel C. (2003). GDNF and TGF-beta1 promote cell survival in serum-free cultures of primary rat microglia. *Cell Tissue Res.* 312 135–139. 10.1007/s00441-003-0711-7 12712323

[B17] Shechter R. Miller O. Yovel G. Rosenzweig N. London A. Ruckh J. (2013). Recruitment of beneficial M2 macrophages to injured spinal cord is orchestrated by remote brain choroid plexus. *Immunity* 38 555–569. 10.1016/j.immuni.2013.02.012 23477737 PMC4115271

[B18] Shechter R. Schwartz M. (2013). Harnessing monocyte-derived macrophages to control central nervous system pathologies: no longer if’ but how’. *J. Pathol.* 229 332–346. 10.1002/path.4106 23007711

[B19] Silver J. Schwab M. E. Popovich P. G. (2015). Central nervous system regenerative failure: role of oligodendrocytes, astrocytes, and microglia. *Cold Spring Harb. Perspect. Biol.* 7:a020602. 10.1101/cshperspect.a020602 25475091 PMC4355267

[B20] Tam W. Y. Ma C. H. E. (2014). Bipolar/rod-shaped microglia are proliferating microglia with distinct M1/M2 phenotypes. *Sci. Rep.* 4:7279. 10.1038/srep07279 25452009 PMC4250916

[B21] Varnum M. M. Ikezu T. (2012). The classification of microglial activation phenotypes on neurodegeneration and regeneration in Alzheimer’s disease brain. *Arch. Immunol. Ther. Exp.* 60 251–266. 10.1007/s00005-012-0181-2 22710659 PMC4429536

[B22] Weber M. S. Prod’homme T. Youssef S. Dunn S. E. Rundle C. D. Lee L. (2007). Type II monocytes modulate T cell-mediated central nervous system autoimmune disease. *Nat. Med.* 13 935–943. 10.1038/nm1620 17676050

[B23] Xing B. Xin T. Zhao L. Hunter R. L. Chen Y. Bing G. (2010). Glial cell line-derived neurotrophic factor protects midbrain dopaminergic neurons against lipopolysaccharide neurotoxicity. *J. Neuroimmunol.* 225 43–51. 10.1016/j.jneuroim.2010.04.010 20471698 PMC2924924

[B24] Yaguchi M. Ohta S. Toyama Y. Kawakami Y. Toda M. (2008). Functional recovery after spinal cord injury in mice through activation of microglia and dendritic cells after IL-12 administration. *J. Neurosci. Res.* 86 1972–1980. 10.1002/jnr.21658 18438913

[B25] Zhang L. Ma Z. Smith G. M. Wen X. Pressman Y. Wood P. M. (2009). GDNF-enhanced axonal regeneration and myelination following spinal cord injury is mediated by primary effects on neurons. *Glia* 57 1178–1191. 10.1002/glia.20840 19170182 PMC2855953

[B26] Zhuravleva M. Rizvanov A. Mukhamedshina Y. (2016). Effect of GDNF on morphology, proliferation, and phagocytic activity of rat neonatal cortex isolated microglia. *Bionanoscience* 6 379–383. 10.1007/s12668-016-0247-4

[B27] Zhuravleva M. N. Mukhamedshina Y. O. Arkhipova S. S. Sanatova E. R. Rizvanov A. A. (2015). The morphological and phenotypic characteristics of microglia at different stages of cultivation and transplantation in the area of spinal cord injury in rats. *Genes Cells* 10 34–39.

